# Case series of outcomes in advanced cancer patients with single pathway alterations receiving N-of-One therapies

**DOI:** 10.1038/s41698-022-00259-7

**Published:** 2022-03-28

**Authors:** Diviya Gupta, Razelle Kurzrock, Suzanna Lee, Ryosuke Okamura, Hyo Jeong Lim, Ki Hwan Kim, Jason K. Sicklick, Shumei Kato

**Affiliations:** 1 School of Medicine, University of California San Diego, La Jolla, CA USA; 2 Center for Personalized Cancer Therapy, UC San Diego Moores Cancer Center, La Jolla, CA USA; 3 Division of Hematology/Oncology, UC San Diego, San Diego, CA USA; 4 Department of Internal Medicine, Veterans Health Service Medical Center, Seoul, Republic of Korea; 5 Division of Hematology and Medical Oncology, Seoul National University Boramae Medical Center, Seoul, Republic of Korea; 6 Department of Surgery, Division of Surgical Oncology, UC San Diego, San Diego, CA USA

**Keywords:** Cancer genomics, Biomarkers

## Abstract

Though advanced cancers generally display complex molecular portfolios, there is a subset of patients whose malignancies possess only one genomic alteration or alterations in one oncogenic pathway. We assess how N-of-One therapeutic strategies impact outcomes in these patients. From 12/2012 to 9/2018, 429 therapy-evaluable patients with diverse treatment-refractory cancers were presented at Molecular Tumor Boards at Moores Cancer Center at UC San Diego. The clinical benefit rate, defined by RECIST1.1, was assessed for patients with solid tumors who underwent next-generation sequencing (NGS) profiling revealing one genomic or pathway alteration, subsequently managed with N-of-One therapies. Nine of 429 patients (2.1%) met evaluation criteria. Using matched therapy indicated by NGS, the clinical benefit rate (stable disease ≥ 6 months/partial/complete response) was 66.7%. Median progression-free survival was 11.3 months (95% CI: 3.4–not evaluable). Thus, a small subset of diverse cancers has single pathway alterations on NGS testing. These patients may benefit from customized therapeutic matching.

Recent advances in precision medicine have quickly transformed treatment strategies for patients with advanced cancers. Currently, genomic testing allows physicians to identify mutated genes and tailor treatment to precisely target the alterations in their malignancies. This approach has been therapeutically beneficial for several cancer types with particular genomic alterations, including BCR-ABL kinase inhibition for chronic myeloid leukemia and KIT inhibition for gastrointestinal stromal tumor (GIST), as well as BRAF and Her2 inhibition for multiple tumor types^1,2^. Furthermore, immunotherapies, such as anti-PD-1/PD-L1 blockades, have shown durable responses among patients with high tumor mutational burden (TMB), microsatellite instability-high (MSI-high), and PD-L1 overexpression/amplification^3–5^.

Despite the identification of numerous biomarkers for targeted therapy, oncology medications have significantly higher drug development attrition rates than medications for non-oncology indications^6^. This is partly because drugs that fail to elicit a durable response in a significant subgroup of patients are frequently abandoned, even if the drug exhibits significant activity in a small proportion of people^7^. Two of the major obstacles to treatment are intra-tumor heterogeneity, in which multiple genomic clonal populations exist within a neoplasm, and inter-tumor heterogeneity, in which multiple tumors in the same patient possess different co-occurring genomic alterations. In fact, patients with advanced cancer harbor a median of five unique oncogenic alterations, suggesting that therapeutics should be individualized and, if indicated, utilize a combination approach^8^. Still, when multiple co-driver alterations exist, it seems likely that patients will exhibit primary or secondary resistance to targeted therapeutic strategies.

Accordingly, we hypothesized that patients whose advanced cancers harbored a single alteration or alterations in a single genomic pathway on interrogation with next-generation sequencing (NGS) would respond especially well to cognate targeted therapy. Herein, we show that such individuals, while uncommon, can often achieve objective and durable responses when administered agents that are well matched to their molecular alteration(s) across a spectrum of cancer types and genomic abnormalities.

Overall, 715 distinct patients with advanced cancer were discussed at face-to-face Molecular Tumor Board (MTB) meetings. Among 429 patients who were subsequently treated and evaluable for outcome analysis, nine patients had a single genomic alteration or alterations in one molecular pathway that were treated with matched targeted therapy (Fig. 1). All nine patients had NGS performed on tissue by Foundation Medicine (FoundationOne™, Cambridge, Massachusetts, http://www.foundationmedicine.com) (Clinical Laboratory Improvement Amendments (CLIA)-certified). The FoundationOne™ tissue assay utilized during the study period interrogated between 182 and 324 cancer-related genes. Median patient age was 41 years (range, 14–72 years). They received a median of two lines of therapy, including matched therapy indicated by NGS results (range, 1–5).

**Fig. 1:**
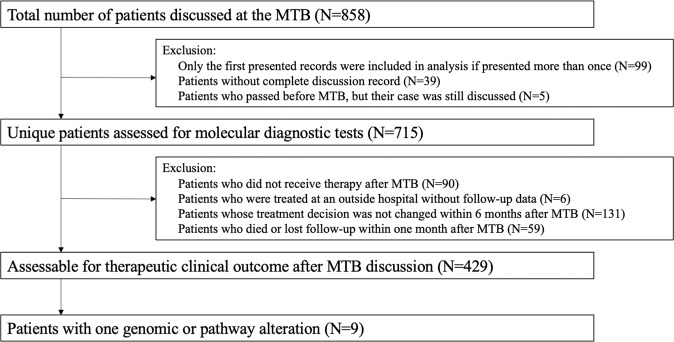
CONSORT diagram of patients included in this study. Only solid tumors were included. Patients who had only one alteration on an initial profiling test but subsequently had more genomic profiling after MTB presentation and demonstrated additional alterations were excluded. All included patients had NGS profiling. Patients were treated within six months of MTB. Patients who received immunotherapy based on MSI-high or TMB-high were not included in the current analysis^9^.

Eight patients received small molecule targeted agent(s), while one patient with *PDL1* amplification received immune checkpoint blockade. Among the nine patients, three patients achieved partial response (PR) and an additional three patients achieved stable disease (SD) ≥ 6 months. The remaining three patients had progressive disease (PD). Altogether, the clinical benefit rate was 66.7% (i.e., 6/9 patients) (Table 1). Median progression-free survival (PFS) was 11.3 months (95% CI: 3.4–not evaluable), while median overall survival (OS) was not reached (95% confidence interval (CI): 8.4–not evaluable) (Fig. 2). Only Patient #5467 (Table 1), who received immunotherapy, experienced a serious adverse event (SAE), namely Grade 3 pancreatitis. No other Grade 3–4 adverse events were observed, according to Common Terminology Criteria for Adverse Events.

**Table 1:** Nine patients who received targeted agents with complete matching.

Study ID	Age	Sex	Diagnosis	Genomic alteration(s) and impact	Regimen and mechanism	Line of therapy	PFS (mos)	OS (mos)	Best response
1192	65	F	Colonic gastrointestinal stromal tumor	*KIT* Y503_F504insAY (Activates KIT kinase)	Sunitinib (multi-kinase KIT inhibitor)	2nd line	2.0	2.0	PD
407	49	M	Medullary thyroid carcinoma	*RET* E632_L633del (Activates RET kinase)	Cabozantinib, then Vandetanib (multi-kinase RET inhibitors)	1st line	3.4	3.4	PD
1421^a^	43	F	Ovarian undifferentiated neuroendocrine carcinoma	*CDKN2A/B* loss (Increases CDK4/6)	Palbociclib (CDK4/6 inhibitor)	3rd line	8.4	8.4	PR
261	72	M	Papillary thyroid carcinoma	*RET-CCDC6* fusion (Activates RET kinase)	Vandetanib (multi-kinase RET inhibitor)	2nd line	35.9	40.1+	PR
1876	14	F	Osteosarcoma	*CCND3* amplification, *CDKN2A/B* loss (Increases CDK4/6)	Palbociclib (CDK4/6 inhibitor)	2nd line	2.2	10.7	PD
2611	33	F	Intrahepatic cholangiocarcinoma	*FGFR2-BICC1* fusion (Activates FGFR2 kinase)	Infigratinib (FGFR inhibitor)	2nd line	21.6+	21.6+	SD ≥ 6 mos
3786	21	F	Brain glioma	*FGFR1* K656E (Activates FGFR1 kinase)	Lenvatinib (multi-kinase inhibitor including FGFR)	2nd line	15.1	25.3+	SD ≥ 6 mos
5288	33	F	Ovarian serous carcinoma	*BRAF* V600E (Activates BRAF/MEK pathway)	Dabrafenib (BRAF inhibitor) Trametinib (MEK inhibitor)	5th line	10.7+	10.7+	PR
5467^b^	41	F	Cervical squamous cell carcinoma	Amplification of *CD274* (*PD-L1*) (Inhibits immune response via PD-L1)	Pembrolizumab (anti-PD1 agent)	4th line	11.3	11.6+	SD ≥ 6 mos

*F* female, *M* male, *mos* months, *OS* overall survival, *PD* progressive disease, *PFS* progression-free survival, *PR* partial response, *SD* stable disease.

^a^In addition to tissue genomic profiling, patient received cell-free DNA genomic profiling, which did not reveal additional mutations.

^b^Patient’s tumor also showed high PD-L1 on immunohistochemistry (tumor proportion score 8%); in addition, *PDCD1LG2* (PD-L2) and *JAK2* (both on the same amplicon as the *PDL1* gene and both sensitizing to immunotherapy) were co-amplified.

**Fig. 2:**
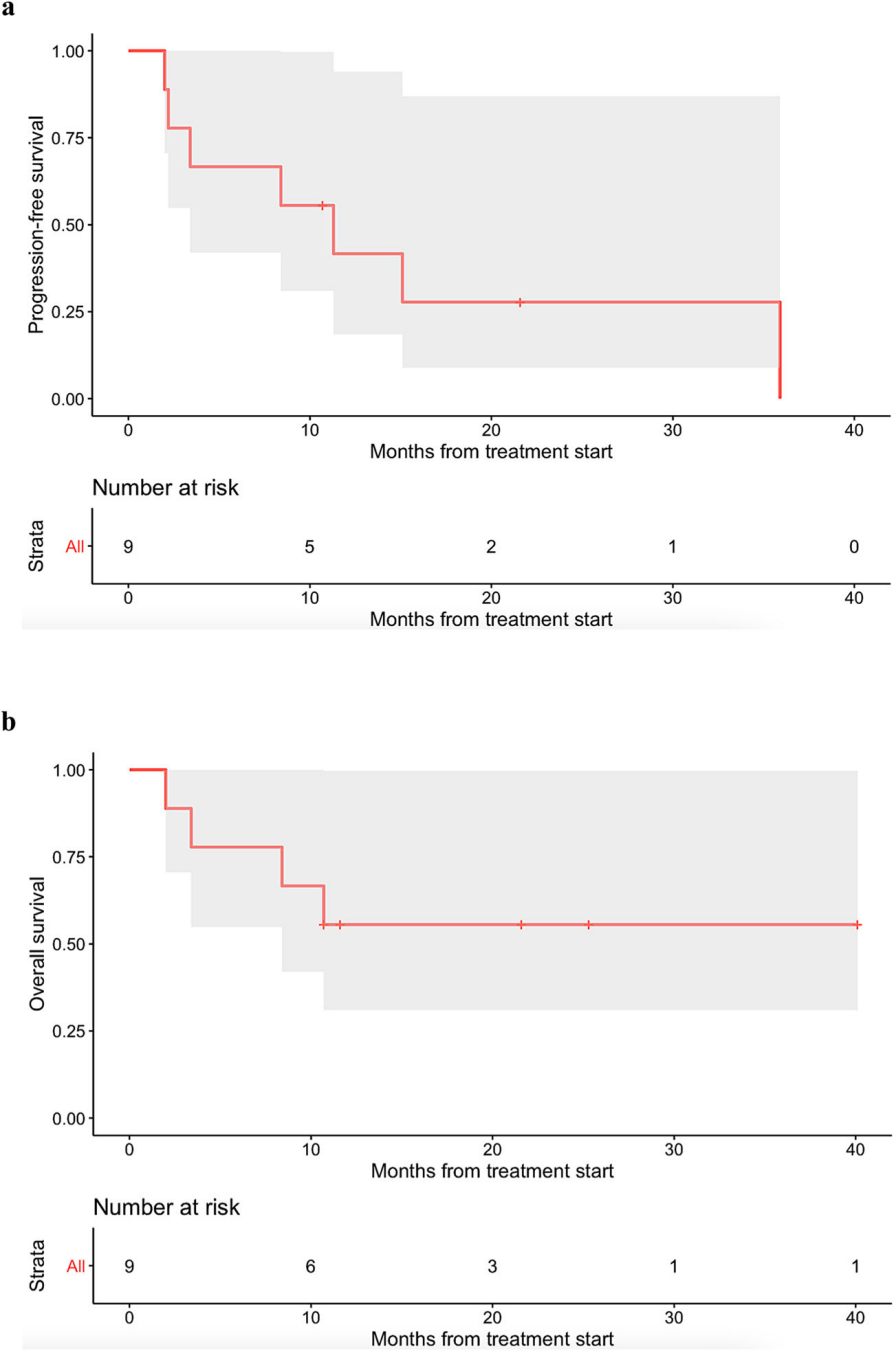
Kaplan–Meier analysis of nine patients with single pathway alterations matched to targeted therapies. The gray shade areas represent the 95% confidence intervals, and bars denote censored observations. Median PFS was 11.3 months (95% CI: 3.4–not evaluable) (**a**), while median OS was not reached (95% CI: 8.4–not evaluable) (**b**).

Recent literature on molecular profiling technologies has revealed that advanced cancer patients harbor a median of five molecular alterations^8^. Although rare, some patients may harbor only one molecular alteration after interrogation of several hundred oncogenic markers with NGS^9^. In this study, the administration of N-of-One treatments was retrospectively reviewed to assess how this approach impacted clinical benefit rates (i.e., PR + SD ≥ 6 months), PFS, and OS in patients harboring one alteration or alterations in one oncogenic pathway.

Overall, 2.1% (9/429) of evaluable patients had one gene/pathway alteration targeted with molecularly matched agents. Six of these nine patients achieved clinical benefit. The pathways successfully targeted included BRAF/MEK, CDK4/6, FGFR, KIT, and RET pathways, as well as PD-L1 amplification-associated immune suppression. Notably, the patient with a *BRAF* V600E mutant ovarian serous carcinoma tumor (i.e., not melanoma) was successfully treated with BRAF inhibitor dabrafenib and MEK inhibitor trametinib (PR ongoing at 10.7+ months). Furthermore, one of two patients with alterations expected to activate CDK4/6 was successfully treated with a CDK4/6 inhibitor as monotherapy (ovarian undifferentiated neuroendocrine cancer, PR lasting 8.4 months); this is in contrast to observations suggesting that matched CDK4/6 inhibitor monotherapy, such as palbociclib, is ineffective^10^, possibly for patients with multiple co-occurring mutations, unlike the patient discussed above. On the other hand, three of the nine patients did poorly. It is plausible that, despite a single pathway alteration on genomics, other important driver pathways were altered at the transcript or protein level in these patients. As such, in addition to tissue genomic profiling, more comprehensive analysis that includes cell-free DNA, transcriptomics, epigenetics, and immune profiling may be considered in the care of future patients.

Limitations of this paper include the retrospective nature of analysis, small sample size, and lack of controls. Despite these limitations, this study provides a window into the opportunity to leverage NGS to prescribe personalized matched therapies for patients with incurable malignancies whose cancers have not yet shown complicated genomic evolution. Trials such as NCI-MATCH^11^ and MSK-IMPACT^12^ have demonstrated the viability of deploying NGS to triage patients to targeted therapeutics, while I-PREDICT^8^ and WINTHER^13^ have evaluated the outcomes of patients receiving therapies that prioritize combination therapy matched to complex genomic and/or transcriptomic profiles. The current study suggests that there is a small subset of patients (~2%) with advanced metastatic disease whose tumors still demonstrate only single pathway alterations on NGS, and that such cancers remain amenable to focused pathway targeting.

## Methods

### Patient selection

We investigated the molecular profiling status (performed by CLIA-certified laboratories) and clinical outcomes of patients with advanced cancer presented at MTB meetings from December 2012 to September 2018, following guidelines of the institutional review board-approved Profile Related Evidence Determining Individualized Cancer Therapy (PREDICT) study (NCT02478931; ClinicalTrials.gov; Posted June 23, 2015) and any investigational therapies for which patients gave consent. Weekly in-person MTBs were held at the Moores Cancer Center at UC San Diego (UCSD) Health and followed protocols as previously described^14^. We studied patients with solid tumors harboring one genomic or pathway alteration managed with matched targeted therapy^8,15^. We excluded patients who received immunotherapy based on MSI-high or TMB-high. However, patients treated with checkpoint blockade were included if the agent targeted discrete alterations such as *PD-L1* amplification^4^. Patients who had only one alteration on an initial profiling test but subsequently received additional NGS profiling that revealed further mutations after MTB discussions were excluded from this analysis.

### Endpoints and statistics

In accordance with RECIST 1.1 criteria, all patients were assessed with the outcome endpoints of clinical benefit rate [i.e., stable disease (SD) ≥ 6 months, partial response (PR), or complete response (CR)] as determined by the treating physician. Median PFS and median OS were also evaluated by the Kaplan–Meier method. PFS was defined as the time from the start of therapy to disease progression or last follow-up date if progression-free (the latter being censored). OS was defined as the time from the start of therapy to death or last follow-up if alive (the latter being censored).

### Declaration of ethical approval

This retrospective case series involves patients enrolled in the UCSD Study of Profile Related Evidence Determining Individualized Cancer Therapy (PREDICT). This study was performed in accordance with UCSD IRB guidelines, and for any investigational treatments for which patients gave consent. All patients underwent informed consent and signed consented forms in their native languages via licensed medical interpreters.

### Reporting summary

Further information on research design is available in the Nature Research Reporting Summary linked to this article.

## Supplementary information

REPORTING SUMMARY

## Data Availability

De-identified data will be made available on reasonable request. Should qualified researchers contact the corresponding author for the de-identified dataset, they will not need to obtain ethical approval or sign a data usage agreement as patient confidentiality will be maintained.
