# Supplementary material for: Case series of outcomes in advanced cancer patients with single pathway alterations receiving N-of-One therapies
Source: NPJ Precis Oncol. 2022 Mar 28;6:18. doi: 10.1038/s41698-022-00259-7 (PMC8960821; doi:10.1038/s41698-022-00259-7)
Supplement: Supplementary file 1 — REPORTING SUMMARY [file 41698_2022_259_MOESM1_ESM.pdf]

nature portfolio

Corresponding author(s): Razelle Kurzrock, MD
Shumei Kato, MD
Diviya Gupta, MD Candidate

Last updated by author(s): 11/15/2021

Reporting Summary

Nature Portfolio wishes to improve the reproducibility of the work that we publish. This form provides structure for consistency and transparency in reporting. For further information on Nature Portfolio policies, see our Editorial Policies and the Editorial Policy Checklist.

Statistics

For all statistical analyses, confirm that the following items are present in the figure legend, table legend, main text, or Methods section.

n/a	Confirmed	
☐	☒	The exact sample size (*n*) for each experimental group/condition, given as a discrete number and unit of measurement
☐	☒	A statement on whether measurements were taken from distinct samples or whether the same sample was measured repeatedly
☐	☒	The statistical test(s) used AND whether they are one- or two-sided<br>*Only common tests should be described solely by name; describe more complex techniques in the Methods section.*
☒	☐	A description of all covariates tested
☒	☐	A description of any assumptions or corrections, such as tests of normality and adjustment for multiple comparisons
☒	☐	A full description of the statistical parameters including central tendency (e.g. means) or other basic estimates (e.g. regression coefficient) AND variation (e.g. standard deviation) or associated estimates of uncertainty (e.g. confidence intervals)
☒	☐	For null hypothesis testing, the test statistic (e.g. *F*, *t*, *r*) with confidence intervals, effect sizes, degrees of freedom and *P* value noted<br>*Give P values as exact values whenever suitable.*
☒	☐	For Bayesian analysis, information on the choice of priors and Markov chain Monte Carlo settings
☒	☐	For hierarchical and complex designs, identification of the appropriate level for tests and full reporting of outcomes
☒	☐	Estimates of effect sizes (e.g. Cohen's *d*, Pearson's *r*), indicating how they were calculated

Our web collection on statistics for biologists contains articles on many of the points above.

Software and code

Policy information about availability of computer code

Data collection: Excel was used for data collection.

Data analysis: R Studio was used for generation of the study image.

For manuscripts utilizing custom algorithms or software that are central to the research but not yet described in published literature, software must be made available to editors and reviewers. We strongly encourage code deposition in a community repository (e.g. GitHub). See the Nature Portfolio guidelines for submitting code & software for further information.

Data

Policy information about availability of data

All manuscripts must include a data availability statement. This statement should provide the following information, where applicable:

- Accession codes, unique identifiers, or web links for publicly available datasets
- A description of any restrictions on data availability
- For clinical datasets or third party data, please ensure that the statement adheres to our policy

The datasets for this study are not publicly published due to presence of potential patient-identifiable information but will be made available from the corresponding author on reasonable request.

Field-specific reporting

Please select the one below that is the best fit for your research. If you are not sure, read the appropriate sections before making your selection.

☒ Life sciences ☐ Behavioural & social sciences ☐ Ecological, evolutionary & environmental sciences

For a reference copy of the document with all sections, see nature.com/documents/nr-reporting-summary-flat.pdf

Life sciences study design

All studies must disclose on these points even when the disclosure is negative.

Sample size	Overall, 715 distinct patients with advanced cancer were discussed at face-to-face Molecular Tumor Board (MTB) meetings. Among 429 patients who were subsequently treated and evaluable for outcome analysis, nine patients had a single genomic alteration or alterations in one molecular pathway that were treated with matched targeted therapy.
Data exclusions	We studied patients with solid tumors harboring one genomic or pathway alteration managed with matched targeted therapy. We excluded patients who received immunotherapy based on MSI-high or TMB-high. However, patients treated with checkpoint blockade were included if the agent targeted discrete alterations such as PD-L1 amplification. Patients who had only one alteration on an initial profiling test but subsequently received additional NGS profiling that revealed further mutations after MTB discussions were excluded from this analysis.
Replication	Findings cannot be reproduced due to the unique patient population in our study.
Randomization	Randomization was not relevant to our study, as our investigation is observational.
Blinding	Blinding was not relevant to our study, as our investigation is observational.

Reporting for specific materials, systems and methods

We require information from authors about some types of materials, experimental systems and methods used in many studies. Here, indicate whether each material, system or method listed is relevant to your study. If you are not sure if a list item applies to your research, read the appropriate section before selecting a response.

Materials & experimental systems

n/a	Involved in the study	
☒	☐	Antibodies
☒	☐	Eukaryotic cell lines
☒	☐	Palaeontology and archaeology
☒	☐	Animals and other organisms
☐	☒	Human research participants
☐	☒	Clinical data
☒	☐	Dual use research of concern

Methods

n/a	Involved in the study	
☒	☐	ChIP-seq
☒	☐	Flow cytometry
☒	☐	MRI-based neuroimaging

Human research participants

Policy information about studies involving human research participants

Population characteristics	Nine of 429 patients (2.1%) met evaluation criteria. There were seven women; median age was 41 years. Cancer types included ovarian (N=2), thyroid (N=2), and one each of glioma, cholangiocarcinoma, cervical squamous cell carcinoma, colonic gastrointestinal stromal tumor, and osteosarcoma. Median lines of systemic treatment, including matched therapy indicated by NGS, was two.
Recruitment	Per Study Protocol:<br><br>Patients will be identified from those having a diagnosis of cancer or cancer-related condition in their medical record seen at a UCSD Health System facility, EMC, or RCHSD prior to the release date of this study's initial IRB approval.<br>A request to waive the requirement to obtain informed consent is being requested for the retrospective chart review data collection and analysis. The investigator believes this part of the study meets the following requirements for this request per 46 CFR 46.116:<br>1. The research involves no more than minimal risk to the participants.<br>2. The waiver or alteration will not adversely affect the rights and welfare of the participants.<br>3. The research could not practicably be carried out without the waiver or alteration. These patients have already been treated.<br>4. Whenever appropriate, the participants will be provided with additional pertinent information after participation.<br>A waiver of HIPAA Authorization is being requested for this retrospective portion of the study. The investigator believes this

	study meets the following requirements for this request per 46 CFR 164. 512(i)(2)(ii):<br>A. The use or disclosure of protected health information (PHI) involves no more than minimal risk to the privacy of individuals;<br>B. The project could not practicably be conducted without a waiver; and<br>C. The project could not practicably be conducted without use of PHI.<br>Further, the privacy risks are reasonable relative to the anticipated benefits of research, as the importance of the knowledge that may reasonably be expected to result outweighs the minimal risk posed to subjects. Section 14, Risk Management Procedures, of the Research Plan includes an adequate plan to protect identifiers from improper use and disclosure and justification for retaining identifiers. PHI will not be re-used or disclosed for other purposes and, whenever appropriate, the subjects will be provided with additional pertinent information after participation. Identifiers will be destroyed by the PI at the completion of the research. Only de-identified data will be kept.
Ethics oversight	UCSD Human Research Protections Program

Note that full information on the approval of the study protocol must also be provided in the manuscript.

Clinical data

Policy information about clinical studies

All manuscripts should comply with the ICMJE guidelines for publication of clinical research and a completed CONSORT checklist must be included with all submissions.

Clinical trial registration	NCT02478931
Study protocol	Study of Personalized Cancer Therapy to Determine Response and Toxicity (UCSD_PREDICT)
Data collection	Patients with diverse treatment-refractory cancers were presented at Molecular Tumor Boards at UCSD during 12/2012 to 9/2018 were reviewed.
Outcomes	Nine of 429 patients (2.1%) met evaluation criteria per the study design. Using matched therapy indicated by NGS, the clinical benefit rate (stable disease ≥6 months/partial/complete response) was 66.7%. Median progression-free survival was 11.3 months (95% CI: 3.4–not evaluable).
